# Editorial: Nucleic acid-based aptamers in therapeutics and diagnostics

**DOI:** 10.3389/fchem.2023.1355870

**Published:** 2024-01-03

**Authors:** Anna Aviñó, Carme Fabrega, Raimundo Gargallo, Stefania Mazzini, Claudia Riccardi

**Affiliations:** ^1^ Department of Surfactants and Nanobiotechnology, Institute for Advanced Chemistry of Catalonia, Spanish National Research Council (CSIC), Barcelona, Catalonia, Spain; ^2^ Department of Chemical Engineering and Analytical Chemistry, University of Barcelona, Barcelona, Spain; ^3^ Department of Food, Environmental and Nutritional Sciences (DEFENS), University of Milan, Milan, Lombardy, Italy; ^4^ Department of Chemical Sciences, University of Naples Federico II, Naples, Campania, Italy

**Keywords:** aptamers, diagnostics, therapeutics, SELEX, nucleic acids (DNA and RNA)

Aptamers have emerged as versatile and powerful tools in the fields of therapeutics and diagnostics. These molecules are single-stranded nucleic acids—either DNA or RNA—that fold into unique three-dimensional structures, allowing them to selectively bind to target molecules with high affinity and specificity. They can be identified through an iterative selection process called SELEX (Systematic Evolution of Ligands by EXponential enrichment).

In the therapeutic domain, aptamers have exhibited tremendous potential, particularly in the development of targeted drug delivery systems. Their ability to specifically recognize and inhibit different type of targets have provided efficient therapeutic payloads directly to the affected cells or tissues, minimizing off-target effects and enhancing the overall treatment efficacy.

On the other hand, aptamers play a pivotal role in diagnostics, offering a robust alternative to traditional detection methods. Their high specificity allows for the precise identification of biomarkers associated with various diseases, facilitating early and accurate diagnosis. Many studies have been carried out using aptamer-based diagnostic platforms based on electrochemical and optical biosensors for the detection of several biomarkers. The rapid and sensitive nature of aptamer-based diagnostics not only expedites the diagnostic process, but also contributes to the development of personalized medicine strategies.

In this context, the aim of the present Research Topic is to highlight recent advances in the use of Nucleic Acid-Based Aptamers in Therapeutics and Diagnostics. Four articles have been selected for publication in this issue, most of which describe diagnostics assays. In their work, Zhdanov et al. developed a Surface-Enhanced Raman Scattering-based aptasensor for the diagnosis of influenza A virus. The proposed methodology, which uses silver nanoparticles, achieves very low detection limits.

The development of a biosensor for the real-time detection of bacteria is the objective of the work by Reed and Gerasimova. The authors proposed a sophisticated strategy based on the use of a fluorescent light-up RNA aptamer. The sensor has been shown to provide excellent results in analyzing folded RNA targets and differentiating between closely related sequences.

In turn, Little et al. proposed the use of another spectroscopy, linear dichroism, for the detection of molecular targets. They described an assay platform for the aptamer-based detection of proteins, such as thrombin.

Finally, the detection of lung cancer typing markers is proposed by Lin et al. This approach, which avoids the complex detection methods of protein markers, is based on a microfluidic chip and a miniaturized nucleic acid analyzer. By combining this experimental procedure with a machine learning model, the classification of lung cancer subtypes was achieved.

In summary, nucleic acid-based aptamers have revolutionized the therapeutic and diagnostic landscape. Their remarkable binding properties, coupled with the ability to target specific molecules, hold great promise for the precise diagnosis and targeted treatment of various diseases. In this context, we hope that the articles collected within the present Research Topic represent a excellent contribution to those fields.

